# Association of single-nucleotide polymorphisms in *SLC2A9, SLC22A12* and *SLC22A11* genes with hyperuricemia in the Chinese Tibetan population

**DOI:** 10.1097/MD.0000000000045023

**Published:** 2025-10-24

**Authors:** Ren-xuan Li, Yu-ning Guo, Jiu Wang, Bu Luo, Guojie Danzen, Tong Zhang

**Affiliations:** a Department of Rheumatology, The Second Affiliated Hospital of Dalian Medical University, Dalian, Liaoning, China; b Department of Radiology, Longhua Hospital, Shanghai University of Chinese Medicine, Shanghai, China; c Department of Tibetan Medicine, Shigatse Tibetan Medical Hospital, Shigatse, Tibet, China; d Department of Urology, Shandong Provincial Hospital Affiliated to Shandong First Medical University, Jinan, Shandong, China.

**Keywords:** ethnic differences, hyperuricemia, serum uric acid, single-nucleotide polymorphisms, Tibetans

## Abstract

Genome-wide association studies have identified a series of genes, including solute carrier family 2 member 9 (*SLC2A9*), solute carrier family 22 member 11 (*SLC22A11*), solute carrier family 22 member 12 (*SLC22A12*) polymorphisms, that are associated with serum uric acid (SUA) levels. The prevalence of hyperuricemia in the Chinese Tibetan population is higher than in other regions of China; however, there is no evidence confirming a genetic association with SUA levels in this population. This study aimed to investigate the association between genetic polymorphisms and SUA levels, as well as hyperuricemia, in a Tibetan population in Tibet, China. A total of 194 Tibetan patients with hyperuricemia and 304 healthy Tibetan controls were enrolled, and polymorphisms in *SLC2A9* (rs1014290), *SLC22A12* (rs559946), and *SLC22A11* (rs1783811) were identified using high-resolution melting. Logistic regression analysis, with adjustments for age and gender, was applied to evaluate the association between genetic polymorphisms and the risk of hyperuricemia, while calculating the corresponding odds ratios (OR) and 95% confidence interval (CI). Linear regression analysis was used to calculate beta values for associations with higher SUA levels. We found no significant association between *SLC2A9* (rs1014290), *SLC22A12* (rs559946), *SLC22A11* (rs1783811), and hyperuricemia in the Tibetan population. Among hyperuricemia patients, *SLC22A12* (rs559946) was negatively correlated with SUA levels after adjusting for gender and age. Under the dominant model, the rs559946 CC genotype was a protective factor against higher SUA levels in hyperuricemia patients (CC vs CT + TT: OR = 0.379, 95% CI: 0.162–0.886, *P* = .025). Under the additive model, rs559946 was also a protective factor (OR = 0.385, 95% CI: 0.175–0.850, *P* = .018). This study is the first to demonstrate that the CC genotype of *SLC22A12* (rs559946) reduces the risk of higher SUA in Chinese Tibetan patients with hyperuricemia.

## 1. Introduction

Uric acid (UA) is the end product of purine metabolism in the human body.^[1]^ Hyperuricemia is primarily caused by elevated purine metabolism or abnormal UA excretion, and is associated with dietary, environmental, and genetic factors.^[2]^ Elevated serum uric acid (SUA) levels have been linked to diseases such as gout, kidney disease, metabolic syndrome, diabetes, hypertension and adverse cardiovascular outcomes.^[3,4]^

The transporters responsible for renal tubular reabsorption are primarily encoded by solute carrier family 22 member 12 (*SLC22A12*) gene, which is predominantly expressed in the kidney and tubular epithelium. Additionally, the solute carrier family 2 member 9 (*SLC2A9*) gene contributes to this process, with widespread expression but primarily in the basal tubules of the kidney. The transporter encoded by solute carrier family 22 member 11 (*SLC22A11*) gene, plays a smaller role in renal tubular reabsorption.^[5]^ Previous genome-wide association studies (GWASs) have identified multiple loci, including *SLC2A9, SLC22A12*, and *SLC22A11*, associated with SUA levels.^[6–8]^

SUA levels are influenced by gene-environment interactions, and genetic effects on UA levels vary across regional populations.^[9,10]^ Studies have explored the relationship between SUA levels and genotype in Asian and Western populations.^[11–13]^ In recent years, the prevalence of hyperuricemia has increased annually, reaching 13.3% in China.^[14]^ However, the prevalence of hyperuricemia among Tibetans in Lhasa, China, is as high as 22.68% – much higher than in some eastern regions and rural areas – suggesting that Tibetans are susceptible to hyperuricemia and highlighting the need for increased attention to its prevention and treatment.^[14–16]^ This study aimed to investigate the association between polymorphisms in *SLC2A9, SLC22A12*, and *SLC22A11* and SUA levels, as well as susceptibility to hyperuricemia, in a Tibetan population in Tibet, China.

## 2. Methods and materials

### 2.1. Study subjects

A total of 498 Tibetan participants (194 hyperuricemia patients and 304 controls with normal UA) were recruited from the hospital between March 2019 and October 2019. All participants were ethnically homogeneous Tibetans from Shigatse, reducing population stratification concerns. The diagnostic criteria for hyperuricemia were SUA levels > 420 µmol/L in men and > 360 µmol/L in women. The prevalence of hyperuricemia increases with age, with a significant rise after 44 years.^[17]^ Participants were categorized into 2 age groups: ≤44 years and >44 years. Higher SUA levels, especially at 535 µmol/L or higher, are associated with an increased risk of gouty arthritis and mortality. I Among hyperuricemia patients, those with SUA > 535 µmol/L were classified into the high SUA group, and those with SUA ≤ 535 µmol/L into the low SUA group.

Exclusion criteria were: acute gouty arthritis or renal calculi; severe liver or kidney disease, malignancy, or other life-threatening conditions; recent use of drugs that affect UA levels such as furosemide, ethambutol; and use of anti-hyperuricemia drugs within 4 weeks prior to the study. Hyperuricemia subjects had no blood relationships, and controls had no personal or family history of hyperuricemia, gout, or other serious diseases. SUA levels and participant characteristics (e.g., gender, age) were recorded. Approval was obtained from the ethics committee of Shandong Provincial Hospital Affiliated to Shandong First Medical University (Grant No. 2020-809). The procedures used in this study adhere to the tenets of the Declaration of Helsinki. All persons gave their informed consent prior to their inclusion in the study.

### 2.2. SNP selection and genotyping

DNA was extracted from whole blood samples using Sequenom MassARRAY® single nucleotide polymorphism (SNP) technology. The average DNA concentration was 50 ± 10 ng/µL, with a stable OD260/280 ratio of 1.8 to 2.0, ensuring that the template quality met the requirements for high-precision genotyping. Candidate genes were selected based on prior research, focusing on those associated with UA excretion: *SLC2A9, SLC22A12*, and *SLC22A11* (all previously linked to hyperuricemia^[3,4,18]^). SNP sites were selected using minor allele frequencies from the International HapMap Project and were accessed through the Database of Genotypes and Phenotypes at the National Center for Biotechnology Information (https://www.ncbi.nlm.nih.gov/gap/). A total of 9 candidate sites were ultimately included: 4 in the non-coding region of *SLC2A9*, 4 in the non-coding region of *SLC22A12*, and 1 in the non-coding region of *SLC22A11* (rs1783811).

### 2.3. Genotyping quality control

The typing call rate is defined as the proportion of successfully typed samples at each locus out of the total number of samples. The average call rate across the 9 loci reached 97.3% (range 95.8–98.5%). Three loci (rs1014290, rs559946, rs1783811) were retained due to their high success rates (98.1%, 97.6%, and 98.0%, respectively) and compliance with Hardy–Weinberg equilibrium (HWE, *P* > .05); the remaining loci were excluded due to call rates <95%. A random sample of 10% (n = 50) was selected for repeat genotyping validation, with an overall error rate of 0.8% (4 inconsistencies out of 500 repeats). No systematic bias was observed, and all inconsistent samples were confirmed by secondary testing. Samples with a missing genotype proportion > 5% (n = 3) were excluded. Gender verification was performed using X chromosome SNPs, confirming no gender classification errors. Amplification and extension primers for each SNP were designed using the online Primer3 software (http://bioinfo.ut.ee/primer3-0.4.0/). SNP genotyping and data analysis were performed using an Automatic Analyzer (Modular 7060, Hitachi, Japan) following standard protocols.

### 2.4. Statistical analysis

Measures with a normal distribution were expressed as mean ± standard deviation (x ± standard deviation), and non-normally distributed measures as median (interquartile range). Student *t* test and rank sum test were used to compare the distribution of normally and non-normally distributed data, respectively. Categorical variables were expressed as n (percentage), and differences between groups were tested using the χ^2^ test. HWE *P*-values for the control group and differences in allele frequencies and genotype distribution between the 2 groups were obtained by χ^2^ test. Linear regression analysis was used to assess the association between gene polymorphisms and SUA values. Logistic regression analysis was used to calculate the odds ratios (ORs) and 95% confidence interval (CI) values to estimate the association between SNPs and the risk of hyperuricemia. All statistical analyses were conducted with SPSS 25.0 (IBM) and PLINK software (version 1.08). Figures were generated using GraphPad Prism 8. A *P*-value <.05 was considered statistically significant.

## 3. Results

### 3.1. Demographic characteristics of participants

Table 1 described the basic demographic characteristics of the participants. There was no difference in the mean age and gender distribution between the hyperuricemia group and the control group, suggesting that the age and gender between 2 groups matched.

**Table 1:** Baseline characteristics of the study patients.

Variable	Hyperuricemia (n = 194)	Control (n = 304)	*P*
Age (yr)	45.87 ± 15.58	45.45 ± 15.17	.764
≤44	91 (46.9%)	142 (46.7%)	.905
>44	103 (53.1%)	162 (53.3%)	
Gender			
Males	122 (62.9%)	177 (58.2%)	.300
Females	72 (37.1%)	127 (41.8%)	
SUA (µmol/L)	459.00 (91)	308.00 (102)	<.001

All continuous variables are expressed as mean ± standard deviation, and categorical variables are expressed as number of cases (percentage).

*P* < .05 indicates statistical significance.

SUA = serum uric acid.

### 3.2. Association of SNPs loci with hyperuricemia

The detailed characteristics of 9 SNPs in the *SLC2A9, SLC22A12*, and *SLC22A11* gene were displayed in Table 2. Three SNPs (rs1014290, rs559946, and rs1783811) were in accordance with HWE (*P* > .05) among healthy controls. Therefore, we conducted genetic association tests only on the congruent 3 gene loci. χ^2^ tests for allele and genotype frequency distributions (Tables 2 and 3) showed no significant differences between hyperuricemia patients and healthy controls for the 3 SNPs (*P* > .05). Stratified analyses by age and gender (Tables S1 and S2, Supplemental Digital Content, https://links.lww.com/MD/Q283) also revealed no association between SNP genotypes and hyperuricemia risk (*P* > .05).

**Table 2:** Allele frequency distribution and association with the hyperuricemia risk.

Gene/SNP	CHR	Position	Allele	MAF		HWE *P*	OR (95% CI)	*P*	
				Hyperuricemia	Control				
*SLC2A9*									
rs1014290	4	10000237	C/T	0.433	0.393	.15	1.18 (0.91–1.53)		.212
rs10489070	4	10274728	G/C	0.302	0.247	4.91E^−11^	1.32 (0.99–1.75)		.057
rs2241480	4	10088139	T/C	0.490	0.477	3.46E^−4^	1.05 (0.82–1.36)		.695
rs3733591	4	9920506	C/T	0.474	0.493	1.86E^−11^	0.93 (0.72–1.20)		.555
*SLC22A12*									
rs559946	11	64591133	T/C	0.214	0.222	1	0.95 (0.70–1.30)		.762
rs7929627	11	64600264	G/A	0.492	0.495	.137	0.99 (0.77–1.28)		.931
rs7932775	11	64600390	C/T	0.392	0.357	2.61E^−3^	1.16 (0.89–1.51)		.267
rs893006	11	64598324	A/C	0.191	0.169	.156	1.16 (0.83–1.61)		.391
*SLC22A11*									
rs1783811	11	64565824	A/G	0.412	0.383	1	1.13 (0.87–1.47)		.359

*P* < .05 indicates statistical significance.

CHR = chromosome, CI = confidence interval, HWE = Hardy–Weinberg equilibrium, MAF = minor allele frequency, OR = odds ratio, SLC22A11 = solute carrier family 22 member 11, SLC22A12 = solute carrier family 22 member 12, SLC2A9 = solute carrier family 2 member 9, SNP = single-nucleotide polymorphism.

**Table 3:** Associations between *SLC2A9, SLC22A12* and *SLC22A11* SNPs and hyperuricemia.

SNP	Model	Genotype	Hyperuricemia	Control	OR (95 % CI)	*P*
rs1014290	Co-dominant	TT	70	118	1	
		TC	80	133	1.01 (0.68–1.52)	.947
		CC	44	53	1.40 (0.85–2.30)	.185
	Dominant	TT	70	118	1	
		TC-CC	124	186	1.12 (0.77–1.63)	.540
	Recessive	TT-TC	150	251	1	
		CC	44	53	1.39 (0.89–2.17)	.150
	Log-additive	–			1.16 (0.91–1.48)	.237
rs559946	Co-dominant	CC	123	184	1	
		CT	59	105	0.84 (0.57–1.24)	.385
		TT	12	15	1.20 (0.54–2.64)	.657
	Dominant	CC	123	184	1	
		CT-TT	71	120	0.89 (0.61–1.28)	.520
	Recessive	CC-CT	182	289	1	
		TT	12	15	1.27 (0.58–2.78)	.548
	Log-additive	–			0.96 (0.71–1.29)	.767
rs1783811	Co-dominant	GG	68	115	1	
		GA	92	145	1.07 (0.72–1.60)	.728
		AA	34	44	1.31 (0.76–2.24)	.330
	Dominant	GG	68	115	1	
		GA-AA	126	189	1.13 (0.77–1.64)	.531
	Recessive	GG-GA	160	260	1	
		AA	34	44	1.26 (0.77–2.05)	.361
	Log-additive	–			1.13 (0.87–1.46)	.360

*P* < .05 indicates statistical significance.

CI = confidence interval, OR = odds ratio, SLC22A11 = solute carrier family 22 member 11, SLC22A12 = solute carrier family 22 member 12, SLC2A9 = solute carrier family 2 member 9, SNP = single-nucleotide polymorphism.

### 3.3. Association of SNPs with SUA levels in total population

**Figure 1.**
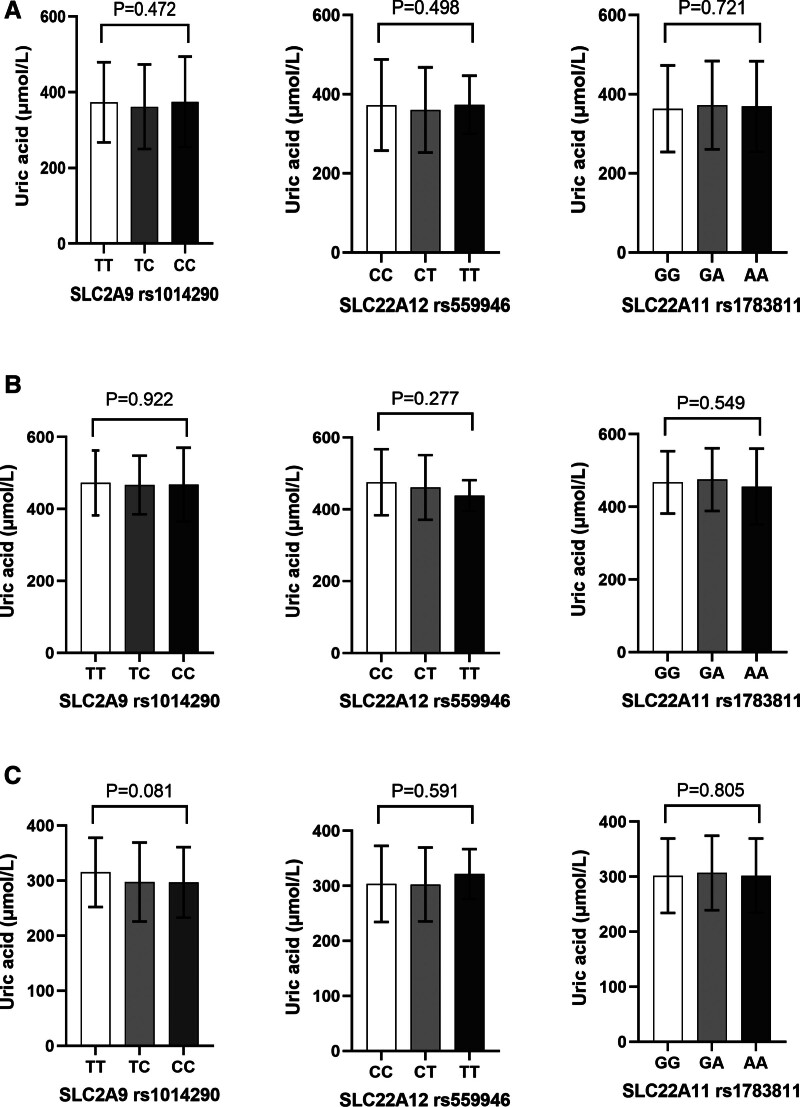
Mean of SUA among the 3 genotypes of SNPs (*SLC2A9* rs1014290, *SLC22A12* rs559946 and *SLC22A11* rs1783811). (A) The SUA were not significantly different across genotypes of rs1014290, rs559946, and rs1783811 in the total population (*P* = .472, *P* = .498, *P* = .721); (B) the SUA were not significantly different across genotypes of rs1014290, rs559946, and rs1783811 in the hyperuricemia groups (*P* = .922, *P* = .277, *P* = .549); (C) the SUA were not significantly different across genotypes of rs1014290, rs559946, and rs1783811 in the control groups (*P* = .081, *P* = .591, *P* = .805). *SLC2A9* = solute carrier family 2 member 9, *SLC22A11* = solute carrier family 22 member 11, *SLC22A12* = solute carrier family 22 member 12, SNP = single nucleotide polymorphism, SUA = serum uric acid. *P* < .05 indicates statistical significance.

As shown in Figure 1, there were no statistically significant differences in SUA levels between individuals with different genotypes of rs1014290, rs559946, and rs1783811 in the total population, hyperuricemia group, or control group. Multiple linear regression analysis and Cochran–Armitage trend tests (adjusted for gender and age) showed no significant correlation between the 3 SNPs and SUA levels in the total population (Table 4). However, in the hyperuricemia group, rs559946 was significantly negatively correlated with SUA levels after adjustment (β = −8.560E^−5^, *P* = .016).

**Table 4:** Association of *SLC2A9, SLC22A12* and *SLC22A11* polymorphisms with SUA levels.

SNP	Total population		Hyperuricemia group		Healthy control group	
	Beta (95% CI)	*P*	Beta (95% CI)	*P*	Beta (95% CI)	*P*
rs1014290	1.840E^−5^ (−0.034, 0.034)	.999	2.308E^−6^ (0, 0)	.936	−48.138 (−98.468, 2.192)	.061
rs559946	−0.007 (−0.049, 0.035)	.743	−8.560E^−5^ (0, 0)	.016	32.337 (−29.189, 93.864)	.302
rs1783811	0.015 (−0.021, 0.052)	.412	4.646E^−6^ (0, 0)	.880	2.473 (−50.502, 55.448)	.927

Evaluation of the association between SNPs and uric acid concentration with linear regression analysis. *P* values were adjusted for age and gender.

*P* < .05 indicates statistical significance.

CI = confidence interval, SNP = single-nucleotide polymorphism, SUA = serum uric acid.

### 3.4. Association of SNPs with SUA levels in a hyperuricemia population

Under the dominant model, the rs559946 CC genotype was a protective factor against higher SUA levels in hyperuricemia patients (GG vs CT-TT, OR = 0.379, 95% CI: 0.162–0.886, *P* = .025). Under the additive model, rs559946 was also a protective factor (OR = 0.385, 95% CI: 0.175–0.850, *P* = .018) (Table 5). These results further indicate that the CC genotype of *SLC22A12* rs559946 reduces the risk of higher SUA levels in hyperuricemia patients.

**Table 5:** Association between *SLC2A9, SLC22A12* and *SLC22A11* polymorphisms and the risk of higher SUA values in hyperuricemia group.

SNP	Model	Genotype	Low SUA	High SUA	OR (95% CI)		*P*
rs1014290	Co-dominant	TT	57	13	1		
		TC	66	14	0.930 (0.404–2.141)	.865	
		CC	36	8	0.974 (0.368–2.582)	.958	
	Dominant	TT	57	13	1		
		TC-CC	102	22	1.038 (0.510–2.113)	.919	
	Recessive	TT-TC	123	27	1		
		CC	36	8	1.245 (0.547–2.833)	.601	
	Log-additive	-			1.086 (0.683–1.728)	0.727	
rs559946	Co-dominant	CC	95	28	1		
		CT	52	7	0.457 (0.187–1.117)	.081	
		TT	12	0	0	.071	
	Dominant	CC	95	28	1		
		CT-TT	64	7	0.379 (0.162–0.886)	.025	
	Recessive	CC-CT	147	35	1		
		TT	12	0	7.711E^−09^ (0)	.997	
	Log-additive	–			0.385 (0.175–0.850)	.018	
rs1783811	Co-dominant	GG	57	11	1		
		GA	74	18	1.260 (0.552–2.878)	.582	
		AA	28	6	1.110 (0.372–3.312)	.851	
	Dominant	GG	74	18	1		
		GA-AA	85	17	1.302 (0.622–2.723)	.484	
	Recessive	GG-GA	131	29	1		
		AA	28	6	1.124 (0.450–2.803)	.803	
	Log-additive	–			1.167 (0.715–1.905)	.536	

*P* < .05 indicates statistical significance.

CI = confidence interval, OR = odds ratio, SNP = single-nucleotide polymorphism, SUA = serum uric acid.

## 4. Discussion

In this case-control study, we investigated for the first time the association between rs1014290 (*SLC2A9*), rs559946 (*SLC22A12*), and rs1783811 (*SLC22A11*) with SUA levels and hyperuricemia in a Tibetan population. Overall analysis showed no differences in allele or genotype frequencies of these SNPs between hyperuricemia patients and healthy controls. Stratified analyses by age and gender also revealed no associations with hyperuricemia risk. However, further stratified analyses showed that *SLC22A12* rs559946 was negatively associated with SUA levels in hyperuricemia patients, and the CC genotype reduced their risk of higher SUA levels.

This study is the first to focus on associations between rs1014290, rs559946, and rs1783811 and hyperuricemia risk in the Chinese Tibetan population, and the first to identify an association between rs559946 and elevated SUA levels in hyperuricemia patients. With changes in lifestyle, diet, and population aging, the prevalence of hyperuricemia is increasing annually. Previous studies have suggested that Tibetans, but not Han Chinese, may have protective factors against hyperuricemia,^[15]^ indicating ethnic specificity in hyperuricemia susceptibility among Tibetans.

GWASs and meta-analyses have identified several genes associated with gout susceptibility, including *SLC2A9, SLC22A12*, and *SLC22A11*.^[5,7,8]^ The *SLC2A9* gene encodes glucose transporter 9, a facilitative glucose transporter that also transports fructose and urate. *SLC2A9* is causally associated with UA levels, and inheritance of susceptibility variants can increase gout risk by 30 to 70%.^[19–22]^ Replication studies of the association of *SLC2A9* with hyperuricemia have been performed in Western and Asian countries. In Mexican samples, rs1014290 allele G was negatively associated with SUA levels.^[11]^ In UK, Croatian, and German populations, rs1014290 (*SLC2A9*) was associated with low fractional UA excretion or gout.^[21]^ Significant differences in SUA levels between *SLC2A9* rs1014290 (TT/TG/GG) genes in Japanese samples.^[12]^ In Han Chinese, the CC^[13]^ and GG^[23]^ genotypes of rs1014290 were associated with lower SUA levels, suggesting that *SLC2A9* polymorphisms may protect against hyperuricemia. *SLC22A11* and *SLC22A12* encode renal uric acid transporter proteins (URATs) located on chromosome 11.^[24]^ According to GWASs, SNP rs17300741 of the *SLC22A11* gene was strongly associated with SUA levels.^[25]^ Multiple SNPs of *SLC22A12* were significantly associated with hyperuricemia and gout.^[26]^ In Han Chinese men, the *SLC22A12* rs559946 polymorphism was associated with hyperuricemia risk, and the major C allele with increased gout risk.^[27]^

In summary, all 3 SNPs were correlated with the development of hyperuricemia, for example, rs1014290 was negatively correlated with hyperuricemia, and rs1783811 and rs559946 were positively correlated with hyperuricemia. However, in the Tibetan population, our study found no association between rs1014290 (*SLC2A9*), rs559946 (*SLC22A12*), rs1783811 (*SLC22A11*), and hyperuricemia. The etiology of hyperuricemia is influenced by environmental factors and lifestyle habits. The lifestyle in Tibetan areas is different from that of mainland China and coastal areas. Tibetan people mostly live in high altitude areas with limited food resources and insufficient food diversity, and Tibetans eat fewer beans than other ethnic groups including the Yi, Han and Zhuang.^[15]^ Tibetans typically consume milk tea, mushrooms, and yak meat. Additionally, due to the high-altitude and alpine climate, alcohol consumption is common in the region as a means of keeping warm and entertaining guests.^[28]^ Notably, certain foods such as yak meat, alcohol, and mushrooms are rich in purines, which may elevate the risk of hyperuricemia.^[29]^ Previous studies have highlighted ethnic differences in hyperuricemia and gout risk.^[30]^ In addition, many studies support the strong presence of familial aggregation of gout.^[31]^ The Tibetan population has lived on the plateau for generations and has a different genetic background from the mainland, with lower levels of heterozygosity and higher levels of purity.^[32]^

Interestingly, we are the first to find that the *SLC22A12* rs559946 C allele may protect Tibetan hyperuricemia patients from higher SUA levels. This finding emphasizes the importance of targeting higher SUA levels: cumulative gouty arthritis incidence reaches 22% within 5 years when SUA ≥ 535 µmol/L,^[33]^ and Irish men with SUA > 535 µmol/L have the highest mortality risk, with an average 11.7-year reduction in life expectancy.^[34]^ Thus, we used 535 µmol/L as the cutoff for higher hyperuricemia. The protective effect of the rs559946 CC genotype against high SUA levels carries important clinical implications for hyperuricemia management in Tibetans. First, this variant’s association with lower SUA suggests its potential role in reducing gout risk. Second, since elevated SUA is linked to cardiovascular and renal comorbidities,^[35]^ the CC genotype may indirectly mitigate these risks. For clinical practice, screening for rs559946 could stratify Tibetans into: CC genotype carriers who may require less intensive interventions, and CT/TT individuals who could benefit from early lifestyle modifications (e.g., purine-restricted diets) or targeted URAT1 inhibition.^[36]^ However, prospective studies are needed to validate its utility in precision prevention. And the rs559946 (*SLC22A12*) deserves more attention from researchers with interest in this field to provide more theoretical basis for targeting interventions at this locus for the treatment of hyperuricemia.

Notably, the pathogenesis of hyperuricemia exhibits significant ethnic disparities.^[37]^ Genetic variants, including SNPs, can alter the function of urate transporter proteins, resulting in different prevalence of hyperuricemia and gout in different populations.^[38]^ For instance, the rs742132 polymorphism of the leucine rich repeat containing 16A gene is significantly associated with hyperuricemia in the Japanese population, yet no such correlation has been observed in Han Chinese males.^[39,40]^ Similarly, the G allele of rs2231142 (ATP binding cassette subfamily G member 2) acts as a protective factor in Uyghur patients with hyperuricemia in China, but shows no association with hyperuricemia in Han Chinese females.^[41]^ Such ethnic differences in disease susceptibility are not unique to hyperuricemia; they have also been observed in other conditions. For example, the low density lipoprotein receptor rs688 polymorphism is significantly associated with ischemic stroke in the Han Chinese population, but not in the Uyghur population.^[42]^

This study has several non-negligible limitations. First, the sample size was relatively small, and thus the findings require validation in a larger Tibetan cohort. Second, although this study aimed to explore the impact of genetic SNP polymorphisms on SUA levels, data on certain risk factors (e.g., smoking, alcohol consumption, and pollution) as well as other metabolic parameters were lacking. Third, despite matching cases and controls by ethnicity and confirming HWE, future studies should incorporate principal component analysis to formally evaluate population structure. Finally, this research is merely preliminary, and further experiments are warranted to investigate the underlying gene expression and protein function.

## Acknowledgments

The authors acknowledge all the physicians and nurses taking part in the enrollment of participants in Shigatse Tibetan Medical Hospital.

## Author contributions

**Conceptualization:** Tong Zhang.

**Data curation:** Yu-Ning Guo.

**Formal analysis:** Ren-Xuan Li.

**Funding acquisition:** Tong Zhang.

**Investigation:** Yu-Ning Guo.

**Methodology:** Tong Zhang.

**Project administration:** Tong Zhang.

**Software:** Ren-Xuan Li.

**Supervision:** Jiu Wang, Bu Luo, Guojie Danzen.

**Validation:** Ren-Xuan Li.

**Visualization:** Jiu Wang, Bu Luo, Guojie Danzen.

**Writing – original draft:** Ren-Xuan Li.

**Writing – review & editing:** Tong Zhang.

## Supplementary Material


