# Supplementary material for: Association of single-nucleotide polymorphisms in SLC2A9, SLC22A12 and SLC22A11 genes with hyperuricemia in the Chinese Tibetan population
Source: Medicine (Baltimore). 2025 Oct 24;104(43):e45023. doi: 10.1097/MD.0000000000045023 (PMC12558201; doi:10.1097/MD.0000000000045023)
Supplement: Supplementary file 1 [file medi-104-e45023-s001.docx]

Supplementary table 1. Association among *SLC2A9*、*SLC22A12* and *SLC22A11* polymorphisms and the hyperuricemia risk stratified by gender.

| SNP | Model | Genotype | Male | | Female | | |
| --- | --- | --- | --- | --- | --- | --- | --- |
|  |  |  | *OR* (95% *CI*) | *P* | *OR* (95% *CI*) | | *P* |
| rs1014290 | Co-dominant | TT | 1 | | 1 | | |
|  |  | TC | 0.994(0.594-1.664) | 0.982 | 1.030(0.506-2.100) | 0.935 | |
|  |  | CC | 1.346(0.709-2.554) | 0.362 | 0.914(0.374-2.232) | 0.843 | |
|  | Dominant | TT | 1 | | 1 | | |
|  |  | TC-CC | 1.131(0.705-1.813) | 0.609 | 1.195(0.647-2.206) | | 0.569 |
|  | Recessive | TT-TC | 1 | | 1 | | |
|  |  | CC | 1.318(0.734-2.366) | 0.355 | 1.522(0.757-3.06) | | 0.239 |
|  | Log-additive | - | 1.142(0.835-1.560) | 0.407 | 1.231(0.828-1.83) | | 0.305 |
| rs559946 | Co-dominant | CC | 1 | | 1 | | |
|  |  | CT | 0.924(0.558-1.529) | 0.757 | 1.433(0.715-2.871) | | 0.309 |
|  |  | TT | 0.819(0.309-2.170) | 0.687 | 0.782(0.185-3.304) | | 1 |
|  | Dominant | CC | 1 | | 1 | | |
|  |  | CT-TT | 1.07(0.665-1.723) | 0.780 | 0.664(0.363-1.212) | | 0.182 |
|  | Recessive | CC-CT | 1 | | 1 | | |
|  |  | TT | 1.165(0.446-3.042) | 0.755 | 1.447(0.376-5.569) | | 0.591 |
|  | Log-additive | - | 1.069(0.731-1.564) | 0.729 | 0.784(0.471-1.307) | | 0.351 |
| rs1783811 | Co-dominant | GG | 1 | | 1 | | |
|  |  | GA | 1.184(0.714-1.964) | 0.512 | 0.921(0.454-1.869) | | 0.819 |
|  |  | AA | 1.030(0.492-2.156) | 0.937 | 1.206(0.505-2.877) | | 0.673 |
|  | Dominant | GG | 1 | | 1 | | |
|  |  | GA-AA | 1.115(0.688-1.808) | 0.658 | 1.172(0.645-2.129) | | 0.603 |
|  | Recessive | GG-GA | 1 | | 1 | | |
|  |  | AA | 0.912(0.464 -1.79) | 0.788 | 1.912(0.99-3.938) | | 0.079 |
|  | Log-additive | - | 1.032(0.731-1.457) | 0.857 | 1.299(0.873-1.931) | | 0.197 |

CI = confidence interval, OR = odds ratio, SNP = single-nucleotide polymorphism. *P*<0.05 indicates statistical significance.

Supplementary table 2. Association among *SLC2A9*、*SLC22A12* and *SLC22A11* polymorphisms and the hyperuricemia risk stratified by age.

| SNP | Model | Genotype | ≤44years | | | >44years | |
| --- | --- | --- | --- | --- | --- | --- | --- |
|  |  |  | *OR* (95% *CI*) | *P* | | *OR* (95% *CI*) | *P* |
| rs1014290 | Co-dominant | TT | 1 | | | 1 | |
|  |  | TC | 0.675(0.370-1.234) | | 0.201 | 1.341(0.753-2.387) | 0.319 |
|  |  | CC | 0.913(0.427-1.954) | | 0.815 | 1.406(0.700-2.824) | 0.338 |
|  | Dominant | TT | 1 | | | 1 | |
|  |  | TC-CC | 1.008(0.592-1.716) | | 0.976 | 1.286(0.762-2.173) | 0.346 |
|  | Recessive | TT-TC | 1 | | | 1 | |
|  |  | CC | 1.630(0.853-3.114) | 0.137 | | 1.200(0.643-2.241) | 0.567 |
|  | Log-additive | - | 1.158(0.815-1.645) | 0.414 | | 1.176(0.836-1.655) | 0.352 |
| rs559946 | Co-dominant | CC | 1 | | | 1 | |
|  |  | CT | 0.916(0.520-1.611) | 0.760 | | 0.878(0.495-1.557) | 0.657 |
|  |  | TT | 0.450(0.049-4.137) | 0.805 | | 1.552(0.632-3.808) | 0.335 |
|  | Dominant | CC | 1 | | | 1 | |
|  |  | CT-TT | 0.759(0.443-1.299) | 0.314 | | 1.022(0.610-1.710) | 0.936 |
|  | Recessive | CC-CT | 1 | | | 1 | |
|  |  | TT | 2.014(0.441-9.210) | 0.367 | | 1.111(0.438-2.822) | 0.825 |
|  | Log-additive | - | 0.863(0.536-1.389) | 0.543 | | 1.032(0.695-1.531) | 0.877 |
| rs1783811 | Co-dominant | GG | 1 | | | 1 | |
|  |  | GA | 0.784(0.429-1.432) | 0.429 | | 1.528(0.873-2.674) | 0.137 |
|  |  | AA | 0.893(0.417-1.912) | 0.770 | | 1.248(0.547-2.851) | 0.598 |
|  | Dominant | GG | 1 | | | 1 | |
|  |  | GA-AA | 1.038(0.607-1.776) | 0.892 | | 1.253(0.742-2.118) | 0.399 |
|  | Recessive | GG-GA | 1 | | | 1 | |
|  |  | AA | 1.294(0.669-2.503) | 0.444 | | 1.183(0.568-2.466) | 0.653 |
|  | Log-additive | - | 1.096(0.766 -1.57) | 0.615 | | 1.175(0.806-1.712) | 0.401 |

CI = confidence interval, OR = odds ratio, SNP = single-nucleotide polymorphism. *P*<0.05 indicates statistical significance.
